# Supplementary material for: Downward accountability mechanism effectiveness by non-governmental organizations in low- and middle-income countries: A qualitative systematic review
Source: PLoS One. 2025 May 28;20(5):e0324098. doi: 10.1371/journal.pone.0324098 (PMC12118924; doi:10.1371/journal.pone.0324098)
Supplement: Attachment — Submitted filename: Comments.docx [file pone.0324098.s004.docx]

**Comment:** The paper presents an analytical look at a very interesting policy question. The paper needs to be better organized with **major modification**:

Is the discussion of the significant research gaps in the effectiveness of downward accountability (DA) mechanisms among NGOs in LMICs comprehensive and supported by sufficient evidence? These is still a lacking behind the research gap foundation. I would advise the authors to rework on it.

What concerns has been addressed about the transparency of data presented in the article, particularly regarding the selection and exclusion criteria used during the systematic review process? The authors need to identity and explain them in detail.

The themes and codes derived from the qualitative analysis are not clearly defined and consistently applied throughout the article.

The article does not adequately address the practical challenges and barriers to implementing DA mechanisms in NGOs. And the recommendations provided to overcome these challenges are not also properly explained with the right implication to it.

There needs to a balanced discussion on both the strengths and weaknesses of the various accountability mechanisms, such as participation, ownership, and social auditing, which I felt is missing in the paper.

The cultural and social factors that have been included in the research for influencing the effectiveness of DA mechanisms, needs to be sufficiently explored, particularly in the context of different LMICs.

Does the article provide clear criteria for assessing the impact of DA mechanisms on the sustainability of local development initiatives and organizational effectiveness? This question need to be answered for more clarity. Is yes then how?
