# Supplementary material for: Downward accountability mechanism effectiveness by non-governmental organizations in low- and middle-income countries: A qualitative systematic review
Source: PLoS One. 2025 May 28;20(5):e0324098. doi: 10.1371/journal.pone.0324098 (PMC12118924; doi:10.1371/journal.pone.0324098)
Supplement: Attachment — Submitted filename: Response_to_Reviewers_auresp_2.docx [file pone.0324098.s007.docx]

**Response Letter to Review Comments**

Title: Downward accountability mechanism effectiveness by non-governmental organizations in low- and middle-income countries: A qualitative systematic review

Submission/Review ID: PONE-D-24-30081

Corresponding Author: Elizabeth Noble ([ehn3724@nyu.edu](mailto:ehn3724@nyu.edu))

**Reviewer 1 Comment 1:** All comments has been addressed. But the manuscript needs a proofread with a native english person before publishing the final version.

**Author Response to Reviewer 1 Comment 1:** The authors have thoroughly reviewed the manuscript and addressed the suggested edits as indicated by the reviewer. We thank the reviewer for their careful review and believe we have identified and corrected all minor proofreading errors.
